# Angiotensin II, Hypercholesterolemia, and Vascular Smooth Muscle Cells: A Perfect Trio for Vascular Pathology

**DOI:** 10.3390/ijms21124525

**Published:** 2020-06-25

**Authors:** Amanda St. Paul, Cali B. Corbett, Rachael Okune, Michael V. Autieri

**Affiliations:** Department of Physiology, Independence Blue Cross Cardiovascular Research Center, Temple University School of Medicine, Philadelphia, PA 19140, USA; tuj63220@temple.edu (A.S.P.); tuj64501@temple.edu (C.B.C.); tuf93708@temple.edu (R.O.)

**Keywords:** vascular smooth muscle cell, angiotensin II, hypercholesterolemia, hypertension, atherosclerosis, vascular diseases

## Abstract

Cardiovascular disease is the leading cause of morbidity and mortality in the Western and developing world, and the incidence of cardiovascular disease is increasing with the longer lifespan afforded by our modern lifestyle. Vascular diseases including coronary heart disease, high blood pressure, and stroke comprise the majority of cardiovascular diseases, and therefore represent a significant medical and socioeconomic burden on our society. It may not be surprising that these conditions overlap and potentiate each other when we consider the many cellular and molecular similarities between them. These intersecting points are manifested in clinical studies in which lipid lowering therapies reduce blood pressure, and anti-hypertensive medications reduce atherosclerotic plaque. At the molecular level, the vascular smooth muscle cell (VSMC) is the target, integrator, and effector cell of both atherogenic and the major effector protein of the hypertensive signal Angiotensin II (Ang II). Together, these signals can potentiate each other and prime the artery and exacerbate hypertension and atherosclerosis. Therefore, VSMCs are the fulcrum in progression of these diseases and, therefore, understanding the effects of atherogenic stimuli and Ang II on the VSMC is key to understanding and treating atherosclerosis and hypertension. In this review, we will examine studies in which hypertension and atherosclerosis intersect on the VSMC, and illustrate common pathways between these two diseases and vascular aging.

## 1. Introduction

### 1.1. Metabolic Syndrome, Hypertension, and Atherosclerosis

Cardiovascular disease is the leading cause of death in the Western world, with 1 in 5 deaths annually attributed to cardiovascular etiology [1]. Vascular diseases including coronary heart disease, high blood pressure, and stroke account for the majority of all cardiovascular diseases, and are increasing worldwide due to the adoption of a Western diet and more sedentary lifestyle. Metabolic syndrome is a clustering of a number of medical conditions, including obesity, high blood sugar (diabetes), high blood pressure (hypertension) and hypercholesterolemia, leading to vascular occlusion (atherosclerosis). The age dependency of diseases within metabolic syndrome’s prevalence is seen in most populations around the world [2]. Clinically, atherosclerosis and hypertension do not occur independently of each other, but it is clear that they can induce and potentiate the severity of each condition. It may not be surprising that these conditions overlap and exacerbate each other when we consider the many cellular and molecular similarities between them. In this review, we will present the cellular and molecular mechanisms that drive atherosclerosis and hypertension, with emphasis on pathways and molecules that intersect on VSMC.

### 1.2. Vascular Inflammation and Atherosclerosis

Atherosclerosis is a chronic, lipid-driven inflammatory disease of the vascular wall. Oxidized lipids are among the earliest initiating factors for the development of atherosclerosis. Lipid oxidation exposes numerous epitopes on the lipoprotein, and an excess of these oxidized lipids become lodged in the subendothelial space, acting as an antigenic, pro-inflammatory compound [3,4]. Indeed, both early [4,5] and more recent [6] studies demonstrate that oxidized lipids and lipid metabolites are cytotoxic, chemotactic, and apoptotic to VSMC as well as macrophages. Ceramides, for example, are found in atherosclerotic plaque, and are quite bioactive, inciting maladaptive signal transduction pathways leading to cytotoxicity, mitophagy, and apoptosis [7]. Infiltrating immune cells, resident endothelial cells (EC), and vascular smooth muscle cells (VSMC) participate in the vascular response to oxLDL, manifested by the activation of NF-kB, the master pro-inflammatory transcription factor in each of these cell types. Endothelial dysfunction is one of the earliest steps in atherogenesis, and NF-κB activation in EC results in the synthesis of adhesion molecules, which further mediate extravasation of circulating monocytes into the artery, propagating localized vascular inflammation. NF-κB activation also induces chemokine synthesis in EC, which advances inflammatory cell extravasation. Cytokines produced by inflamed EC activate mal-adaptive signaling cascades in VSMC, resulting in the synthesis of cytokines, matrix proteins and matrix degrading enzymes by these cells, resulting in a feed-forward process leading to exacerbation of a localized inflammatory reaction. A number of reviews on the role of EC in atherogenesis have been published; since the aim of this review is a focus on VSMC, we refer the reader to the following excellent reviews [8,9]. VSMC in particular play an important and understudied role in atherosclerosis, and a recent study has suggested that as many as 70% of all cells in atherosclerotic lesions are SMC-derived [10,11]. This information, coupled with the fact that VSMC contractility mediates vascular lumen size, illustrates why VSMC will be the primary cell type focused on in this review. The secretion of proliferative and inflammatory cytokines and immune modulators promulgate autocrine activation of VSMC and further the recruitment of macrophages to the lesion in a paracrine manner [12]. The migration, proliferation, and synthesis of extracellular matrix by VSMC contribute to early formation of lesions [13]. The highly inflammatory lipid core of an atherosclerotic plaque is often contained by a dense cap of activated smooth muscle cells and matrix proteins. Plaque, even advanced plaque, tends to be non-obstructive as the lesion grows outward. The central pathology of advanced plaque is its vulnerability to rupture, resulting in rapid formation of thrombi, acute cessation of coronary perfusion, and tissue ischemia. In this scenario, the proclivity of plaque to rupture is correlated with the structural integrity of its cap, which is directly related to the inflammatory state of the VSMC in the atherosclerotic lesion [14]. In summary, a factor which can activate or enhance an inflammatory response in VSMC may be considered to exacerbate atherosclerosis, and therefore can promote the likelihood of morbidity and mortality.

### 1.3. Hypertension and Angiotensin II

**Figure 1:**
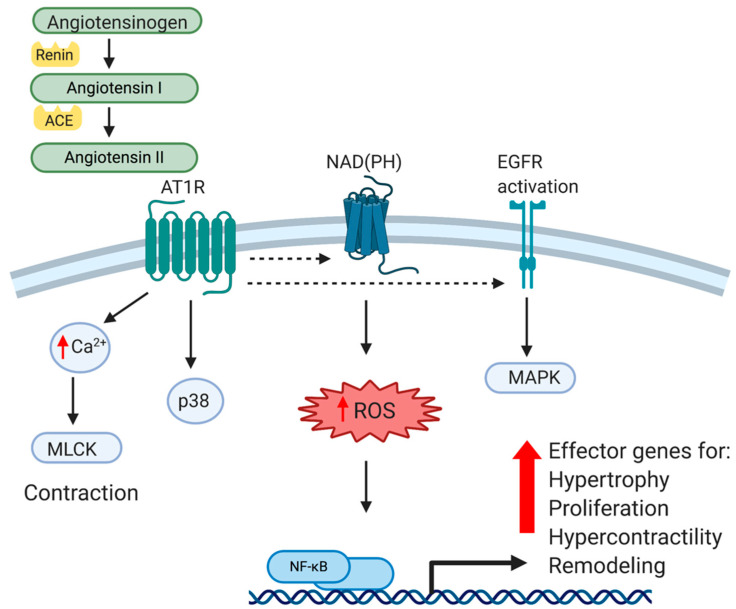
Cellular and Molecular effects of Angiotensin II on VSMC. Angiotensin II (Ang II) is produced from the conversion of angiotensinogen to angiotensin I by renin, and then cleaved by angiotensin-converting enzyme (ACE) into Ang II. Ang II is recognized by a G-protein-coupled receptor, Angiotensin type 1 (AT1R). Canonical GPCR signaling activates receptor tyrosine kinases such as epidermal growth factor receptor (EGFR) as well as activating the NAD(P)H complex, resulting in generation of reactant oxygen species (ROS), a potent second messenger. In addition to regulation of VSMC contractility in a calcium/calmodulin-mediated pathway, AT1R stimulation results in activation of MAPK, and ultimately, NF-κB transactivation. Together, this leads to VSMC pathophysiological responses such as matrix production, hypertrophy, hypercontractility, vascular remodeling and hypertension.

The renin-angiotensin system (RAS) functions in an endocrine fashion to influence numerous organs throughout the body. The RAS plays an essential role in the regulation of blood pressure through the regulation of peripheral vascular resistance and by influencing the electrolyte and fluid balance in the organism. The major effector peptide of this system, angiotensin II (Ang II), is produced from the conversion of angiotensinogen to angiotensin I by renin, which is then cleaved by angiotensin-converting enzyme (ACE). Ang II is recognized by two different G-protein-coupled receptors, Angiotensin type 1 (AT1R) and Angiotensin type 2 (AT2R). Angiotensin type 1 receptors are expressed in most tissue and mediate most of Ang II’s effects, particularly those that are vasoactive. Vascular type-1 and type-2 receptors have opposing effects. While AT1R mediates hypertensive effects, AT2R activation produces hypotension, and has anti-hypertensive effects. AT1R signaling and mediated gene expression will be the major focus of this review. While AT1R is widely expressed in many tissues, we will limit our discussion to that which is reported in VSMC. AT1R is a G-protein coupled receptor (GPCR), and upon ligand binding activates the classical G-protein related pathway. Canonical GPCR signaling regulates VSMC contractility in a calcium/calmodulin-mediated pathway, which activates MLCK activation and actin/myosin interactions to mechanize cellular contraction [15]. This receptor-mediated pathway also activates receptor tyrosine kinases such as epidermal growth factor receptor (EGFR) and platelet derived growth factor receptor (PDGF), among others. Importantly for this review, AT1R activates p38, a MAPK and central integrator of inflammatory signaling [16]. Ang II stimulated MAPK activation is significantly higher in VSMC isolated and cultured from spontaneously hypertensive rats (SHR) compared to normal VSMC, suggesting an intimate relationship between the AT1R and MAPK pathways [17]. One distal event of p38 and receptor tyrosine kinase activation is the activation of transcription factors, most-notably NF-κB, a master regulator of pro-inflammatory gene expression. In cultured VSMC and aorta of animals, Ang II directly activates NF-κB through AT1R [18]. It has long been recognized that in VSMC, Ang II is a powerful activator of oxidant stress, and a second major pathway through which Ang II activates NF-κB is through oxidative stress. Ang II generation of reactant oxygen species (ROS) in a NAD(P)H-dependent mechanism leads to a number of maladaptive cellular responses [19,20]. Although vascular NAD(P)H are essential in the normal physiology of VSMC, they also catalyze excessive ROS (for excellent review, see [21]). ROS in turn, by virtue of being a potent second messenger, activates a number of transcription factors in VSMC, again most notably NF-κB. Pertinent in any discussion of atherosclerosis, this results in maladaptive expression of inflammatory cytokines such as TNFα, chemokines like MCP-1, adhesion molecules such as ICAM1, and matrix and matrix modifying proteinases [22,23,24]. Together, these molecular events act in concert and result in cellular responses such as VSMC hypertrophy, proliferation, oxidation, and altered matrix metabolism, all which lead to classic pathophysiological indices of vascular stiffness, loss of compliance, and remodeling indicative of hypertension. These molecular events are summarized in Figure 1. Importantly, this also leaves the vessel wall primed for inflammatory insult by hypercholesterolemia. Clinically, atherosclerosis and hypertension are considered to be separate diseases with different diagnoses and treatments. This being said, from a molecular, cellular and vascular perspective, there are many similarities, and perhaps too many opportunities for cross-talk between hypertensive and atherogenic stimuli and outcomes. These will be discussed in the next sections.

## 2. Relationships between RAS, Inflammation, and Hyperlipidemia

**Figure 2:**
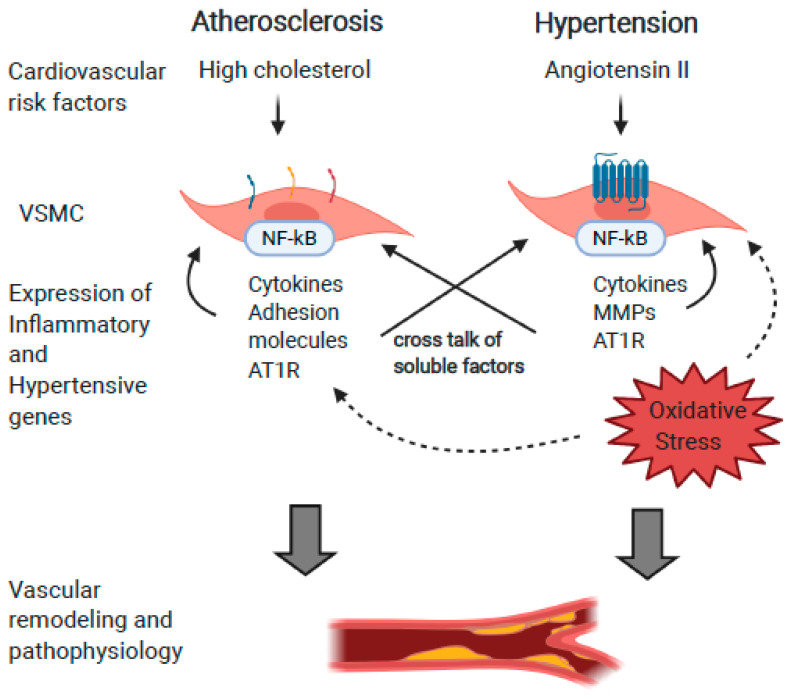
Systemic, cellular, and molecular intersections between hypertension and atherosclerosis. Cardiovascular risk factors such as elevated cholesterol and chronic hypertension converge on VSMC, and activate the expression of RAS components and inflammation-related genes. Soluble factors such as cytokines, as well as oxidative stress activate VSMC in an autocrine and paracrine manner. This results in a vasculature predisposed to inflammatory and hypertensive signals, and thus more vulnerable to atherosclerosis and hypertension. This process is exacerbated in aged individuals, and leads to increased remodeling indicative of advanced age.

It has become evident that there is an intimate relationship between atherogenic and hypertensive factors as protagonists of vascular inflammation and the induction of each other. There is a clear link demonstrating that atherogenic stimuli can induce the expression of components of the RAS, supporting the hypothesis that the RAS is mechanistically pertinent in the development and, potentially, the treatment of atherosclerosis. This is illustrated in Figure 2.

### 2.1. Atherogenic Stimuli Induce Ang II and Components of the RAS Pathway

In VSMC, inflammatory cytokines and other factors can modify AT1R expression. Early studies in New Zealand white rabbits showed that when fed a high cholesterol diet, AT1R, but not AT2R, expression was increased five-fold in medial and intimal VSMC [25]. Similarly, the expression of many RAS components was significantly increased in ApoE(−/−) mice fed a high fat diet (HFD) compared with those fed a chow diet (CD). Explanted aortic rings validated a significantly increased response to Ang II, demonstrating the functional significance of this upregulation. In a similar study, low-density lipoprotein increased expression of the AT1R gene in cultured vascular smooth muscle cells [26]. In this study, myography was used to quantitate contractility in isolated aortic rings. It was concluded that the angiotensin II-induced vasoconstriction in isolated rings from hypercholesterolemic rabbits was significantly increased compared with normocholesterolemic rabbits. This also correlated with a two-fold increase in the density of cell surface AT1R in the aorta of hypercholesterolemic rabbits. In LDLR−/− mice fed a HFD, the angiotensin converting enzyme (ACE) protein was detected in multiple cell types in atherosclerotic lesions [27]. Translating this into humans, it was found that oxidized LDL activated NF-κB, which led to AT1R expression and reduced cell viability in cultured human coronary artery EC [28]. Moreover, in humans, immunohistochemical co-localization determined that Ang II, ACE, and AT1R are all expressed in atherosclerotic plaque in patients with unstable angina [24]. These RAS component proteins were enriched in VSMC in proximity to rupture sites, and co-localized with the pro-inflammatory cytokine IL-6. When taken together, it is clear that the inflammatory cascade initiated by cholesterol and other lipid components predispose aortic VSMC and EC toward enhanced responsiveness to angiotensin II and hypertension.

### 2.2. Angiotensin II Induces Expression of Atherosclerotic Proteins

Angiotensin II can induce atherosclerosis at two levels; the first is systemic, by increasing oxidation and other modifications of circulating LDL. The second is more direct at the cell and molecular level by the AT1R-mediated induction of lipid receptors and pro-inflammatory matrix proteins and matrix-degrading enzymes. Oxidative stress is a major protagonist in atherogenesis and is induced by Ang II. The preeminence of oxidative stress in the development of atherosclerosis is illustrated in a recent study, in which gene expression and mass spectrometry analysis was performed from VSMC isolated by laser-captured microdissection in atherosclerotic and non-atherosclerotic patients [29]. Gene Ontology (GO) analysis identified that the major discriminators between atherosclerotic and non-atherosclerotic patients were those involved with superoxide radical generation, and particularly superoxide dismutase (SOD1) overexpression. An additional protein uncovered in this study was the SERPIN family-related protein SERPINE2, which is note-worthy in that members of the SERPIN family have recently been identified as key regulators of VSMC migration, adhesion, signal transduction and proliferation, linking atherogenic stimuli and VSMC dysfunction [30,31]. Ang II exerts cellular damage via the production of reactive oxygen species in VSMC and its paracrine effects on other cells in proximity in an NAD(P)H-dependent mechanism [32]. 

Because Ang II promotes the generation of oxidative stress in the vasculature, it contributes to lipoprotein modification by peroxidation of LDL [33]. Ang II-induced peroxidation of native LDL leads to its modification, and aggregation of LDL are both risk factors for and major protagonists of atherosclerosis due to their inflammatory properties [34]. Interestingly, a greater proportion of modified LDL was obtained from hypertensive patients compared with normotensive controls [35]. LDL receptor-related protein (LRP1) internalizes cholesteryl esters (CE) from aggregated LDL (agLDL). Ang II-treatment of VSMC increases their expression of LRP1, and Ang II treatment in rats increases LRP1 expression in their aorta, together increasing VSMC foam cell formation and propensity for unstable plaque [36].

Although EC are not the focus of this review, it would be remiss not to mention the profound effects of Ang II on endothelial cells. As the gateway to vascular pathophysiology, the health of the endothelium has profound effects on VSMC and atherogenesis. These effects are both direct via the ATIR, and indirect by increases in lipid oxidation and the generation of intracellular ROS. Treatment of human EC with Ang II was found to induce the expression of the endothelial oxLDL receptor, LOX-1 (lectin-like oxLDL receptor-1) [37]. This increased expression facilitates the increased cellular uptake of oxidized LDL and induces EC dysfunction, one of the initial cellular steps in atherogenesis [38]. This increased LOX-1 expression was inhibited by the AT1 receptor antagonist losartan. Importantly, arterial biopsy samples showed decreased LOX-1 mRNA expression in patients treated with ACE inhibitor therapy, providing a link between Ang II and atherogenesis at the molecular, cellular, and clinical levels. Oxidative stress is a major early risk factor in endothelial dysfunction, and endothelial dysfunction is the initial and obligate step in atherogenesis. Ang II oxidative stress is a major cause of endothelial dysfunction at several levels. Ang II-mediated increase in oxidative stress dysregulates EC production of NO, leading to impaired endothelial relaxation [39]. Moreover, the increase in intracellular ROS induced by Ang II initiates cellular pathways leading to endothelial cell apoptosis and increased endothelial thrombogenicity reviewed in [40]. Oxidative stress not only impairs vascular reactivity and vasodilation [41], but chronic ROS exposure causes EC senescence by a MAPK-dependent mechanism [42]. The consequences of Ang II senescence on the endothelium are reduced EC regenerative capacity and an increase in thrombogenicity due to increased adhesion molecule expression [43]. Because of their interface between the circulation and with medial VSMC, the effects of Ang II on the endothelium, and how this impacts VSMC cannot be overlooked.

Angiotensin II exerts direct atherogenic effects on VSMC at the cell and molecular level via AT1R engagement. Not only can Ang II oxidize excess LDL, a major atherogenic event, but intracellular ROS can act as a potent second messenger [44], leading to inappropriate transcription factor activation and the subsequent maladaptive gene expression of adhesion molecules and pro-inflammatory cytokines in the vessel wall. Many studies using cultured cells demonstrated that the addition of Ang II induced expression of MCP-1, a potent leukocyte chemokine, in a dose-dependent fashion [45]. Importantly, this induction could be inhibited by the AT1R antagonist losartan. IL-18 is a potent pro-inflammatory cytokine, activating NF-κB and inducing the expression of inflammatory cytokines and adhesion molecules in VSMC. Pretreatment of VSMC with Ang II not only potentiated IL-18 induced expression of pro-inflammatory genes, but also increased IL-18 receptor subunit expression, suggesting a feed-forward Ang II/IL-18 pro-inflammatory axis in VSMC [46]. VSMC migrate, proliferate, and increase in size in response to Ang II stimulation, which, in concert with increased matrix synthesis, contributes to vascular lumen occlusion [47]. In both EC and VSMC, Ang II promotes expression of matrix metalloproteinase and plasminogen activator inhibitor-1 [48]. In this manner, Ang II, by virtue of its effects on matrix and MMP expression in VSMC, leads to cap degradation, which promotes plaque vulnerability and rupture, the major cause of morbidity and mortality in humans [49,50,51]. In total, these data paint a picture strongly suggesting that Ang II, at multiple levels, can “prime” the vasculature for the multitude of pro-inflammatory events incited by hypercholesterolemia, leading to atherosclerosis.

## 3. Direct Causative Relationships between Angiotensin II and Atherosclerosis

Clear links between the renin-angiotensin system, angiotensin II signaling, and pathogenesis of atherosclerosis have been reported. In an early study, the relationship between atherosclerosis and hypertension was noted in ApoE−/− mice [52]. ApoE−/− mice develop hypercholesterolemia and subsequent atherosclerotic lesions, and in this study, hypertension was observed in mice that had developed plaque compared with mice which had not. Multiple experimental in vivo approaches including infusion of Ang II and treatment with anti-hypertensive medications into hyperlipidemic animals, and use of genetically modified mice in which components of the RAS have been deleted are supported by clinical studies in humans which confirm the intersection between these two diseases. All four topics will be reviewed.

### 3.1. Angiotensin II Can Exacerbate Vascular Inflammation and Promote Atherosclerosis

Infusion of Ang II in ApoE−/− mice results in accelerated atherosclerosis [53]. In this study, osmotic mini pumps were implanted in ApoE−/− mice to deliver Ang II systemically. As expected, treated mice became hypertensive, but also demonstrated significantly increased atherosclerotic plaque compared with controls. A second study using the same methodology validated these results, and detailed plaque characterization identified lesions with lipid-laden macrophages and increased lymphocyte infiltrate, particularly in the vascular adventitial compartment [54]. Similar to ApoE−/− mice, LDLR−/− mice infused with Ang II also developed increased atherosclerosis. However, this was abrogated when these mice were injected with neutralizing doses of anti-TGFβ antibody [55]. Considering the effects of Ang II on matrix production, this may not be entirely surprising, but it does demonstrate proof of principle in an in vivo model. From a mechanistic standpoint, it is recognized that oxidative stress can induce the deamidation of proteins containing an Asn-Gly-Arg (NGR) motif, which is present on EC integrins [56]. This structural change in EC adhesion molecules results in a gain-of function, leading to increased adhesion with circulating leukocytes [57,58]. While this review focuses on VSMC, this does represent a potentially important way in which Ang II-induced oxidation can amplify its effects of oxidative stress and contribute to leukocyte extravasation and development of atherosclerosis.

### 3.2. Pharmacological Inhibitors of RAS Attenuate Atherosclerosis

Numerous studies in mice indicate that angiotensin converting enzyme (ACE) inhibitors attenuate a variety of cellular events underlying atherogenesis. In earlier studies, ApoE−/− mice treated with antihypertensive drugs had reduced atherosclerotic plaque burden, which the authors attributed to decreased LDL oxidation [59]. Angiotensin converting enzyme (ACE) inhibitors reduced plasma cholesterol, foam cell formation and plaque size in hamsters fed a HFD [60]. In one study, ApoE−/− mice were infused with Ang II, resulting in increased atherosclerosis as well as the expected increase in hypertension. The inclusion of Enalapril, a potent ACE inhibitor, in drinking water significantly reduced atherosclerotic burden in these mice, and importantly, reduced expression of cytokines, adhesion molecules and macrophage infiltrate in the aortic arch of the treated mice [61]. These investigators found that Enalapril up-regulated the expression of the anti-inflammatory transcription factor peroxisome proliferator-activated receptors (PPAR)-gamma, adding mechanistic insight to this study. In one interesting report, a combination strategy was performed in which a statin was administered to hypercholesterolemic rabbits simultaneously with an AT1R blocker [62]. Plaque burden was inhibited more in the combination group compared with the statin alone group, again pointing to Ang II signaling as a pro-atherogenic event.

### 3.3. Studies in Genetically Modified Mice

Numerous studies using genetically modified mice enabled a reductionist approach independent of off-target effects inherent in use of pharmacological inhibitors and, most importantly, generally validated results obtained by pharmacological methods. One demonstration of the rigor of these studies is that they were performed in a number of different animal models. In one study, AT1R knock out mice were crossed with ApoE−/− mice (AT1R/ApoE DKO) [63]. The authors observed that the DKO mice had significantly smaller plaque area compared with control mice. Expression of matrix and matrix metalloproteases in the aorta were also lower, suggesting protective effects for plaque vulnerability. In another study using AT1R/ApoE DKO mice [64], systolic blood pressure was significantly lower in the double knockouts, which was not unexpected. However, while plasma cholesterol remained the same between the test and control groups, the double knockout mice developed significantly less plaque compared to all control groups. Furthermore, treatment of these mice with anti-hypertensive medication lowered blood pressure, but only the administration of an AT1R antagonist significantly reduced plaque. Conversely, a protective role for AT2R in atherosclerosis has been shown, as AT2R/apolipoprotein E (ApoE)–double-knockout (AT2R/ApoE-DKO) mice were shown to have increased plaque burden [65]. Importantly for this discussion, an increase in expression of p47^phox^ and oxidative stress was observed. Treatment of these mice with the AT1R antagonist Valsartan reversed these findings. These data were validated with an over-expression approach, in which AT2R was overexpressed in LDLR−/− mice by AAV transduction [66]. Atherosclerotic plaque was significantly reduced in the AT2R-over-expressing mice compared with controls. Importantly, the expression of vascular protective genes such as eNOS and HO-1 was increased in the AT2R-over-expressing mice. One limitation to many of these studies is that they were performed in mice that were genetically modified to develop hypercholesterolemia, and thus atherosclerotic plaque. In this regard, it cannot be concluded that Ang II alone can induce atherosclerosis in the absence of hypercholesterol, as wild-type mice do not develop atherosclerosis. 

Nevertheless, when taken together, it appears that in the context of a high fat diet, AT receptor activation participates in more than blood pressure regulation, and in the case of AT1R, promotes the pathogenesis of atherosclerosis. This opens up the possibility that AT1R antagonists can be used as an adjunct to cholesterol-lowering therapeutics for the treatment of atherosclerotic vascular disease, a picture which is beginning to emerge in human clinical studies.

### 3.4. Clinical Studies in Humans

Consistent with metabolic syndrome, high serum cholesterol is quite often present in patients with hypertension. Hypertensive patients are more prone to atherosclerotic lesions and acute ischemic events than normotensive individuals, and there is a strong correlation between high plasma cholesterol and impaired endothelium-dependent relaxation [67]. By far, the most common lipid lowering therapies in current use are the HMG-CoA reductase inhibitors, better known as statins. In the liver, HMG-CoA reductase produces mevalonate and is the rate limiting enzyme for cholesterol biosynthesis. Nevertheless, it is well accepted that statins have pleiotropic effects. Anti-inflammatory effects of statins independent of mevalonate synthesis inhibition have been reported and are most likely due to their inhibition of synthesis of isoprenoid intermediates [68]. These isoprenoid intermediates are lipid anchoring points for signal transduction proteins to localize to the plasma membrane, thus potentially attenuating inflammatory signal transduction [69]. Considering what we know about the intersection of AT1R mediated pro-inflammatory signaling and vascular inflammation, it should not be surprising that these drugs are effective in the reduction of hypertension. There are ample clinical data that indicate that statins can decrease hypertension independent of lowering cholesterol. In several clinical studies, the inclusion of a statin, combined with anti-hypertensive medication had synergistic effects on lowering both systolic and diastolic BP. Some statins were found to be effective in reducing hypertension and atherosclerosis either partially or completely, with mechanisms unrelated to their lipid-lowering effects [70,71]. Hypertensive patients with high cholesterol placed on statins demonstrated significantly lower mean arterial BP compared with the control group [72]. In another study, Rosuvastatin demonstrated an additive effect with antihypertensive therapy in lowering BP [73]. One study was designed to investigate the effects of the ACE inhibitor Enalapril and the angiotensin receptor blocker Candesartan on circulating atherosclerotic risk factors such as adhesion molecules and clotting factors in patients with non-insulin-dependent diabetes mellitus (NIDDM) [74]. Each of these drugs were effective in reducing circulating levels of these pro-inflammatory, pro-atherogenic factors. In one double-blind, placebo-controlled study in adults with high-cholesterol, but not hypertension, the multiple statins tested had a moderate but statistically significant reduction in blood pressure [75]. The CORAL study tested the efficacy of combining anti-hypertensive and cholesterol lowering therapy [76]. It was concluded that mean blood pressure decreased significantly more when a statin was included in the anti-hypertensive regimen, and that multiple lipid parameters such as LDL and triglyceride concentrations were significantly decreased when anti-hypertensives were included in the anti-atherosclerotic treatment regimen. From a mechanistic standpoint, increased vascular contractility may result from the known effects of hypercholesteremia on the impairment of nitric oxide biosynthesis [77]. Statins were also shown to increase NO synthesis in EC by post-transcriptional mechanisms, possibly by increasing eNOS mRNA stability [78]. The synthesis of the multitude of studies in which Ang II is infused or the RAS pathway is antagonized by pharmacological inhibition or genetic deletion, together in concert with clinical studies in humans, demonstrate correlative as well as causative effects of the intersection of hypertension and atherosclerosis.

## 4. Angiotensin II, Atherosclerosis, and Vascular Aging

Aging can be considered a vascular disease wherein decades of subclinical inflammatory and oxidative stress often results in arterial wall thickening, stiffening, and vulnerability to hypertension and atherosclerotic plaque development. Interestingly, the pro-inflammatory aging phenotype occurs with little to no mononuclear cell infiltrate, yet the vasculature is predisposed to inflammatory insult. Similarly, the incidence of clinically relevant hypertension increases exponentially with aging. Numerous cytokines such as platelet-derived growth factor (PDGF), matrix-inducing factors such as transforming growth factor-β, matrix proteins, matrix metalloproteinases, and vasoregulatory molecules such as endothelin (ET1) are increased in aged arteries, resulting in characteristic structural changes such as a thickened intima and a disorganized medial compartment [79]. Decades of insult from reactive oxygen species (ROS) lead to profound changes in the endothelium, including EC senescence and apoptosis [80,81]. In the media, histologically, VSMC tend to be less organized, and elastic laminae less regular and fractured. Matrix proteins, particularly the collagens, are produced by and deposited around senescing VSMCs, and together with elastic fiber fragmentation reduce vascular compliance [82]. Arterial stiffening is associated with hypertension, atherosclerosis, and cerebrovascular incidents. In one study, right carotid artery stiffness was quantitated in old versus young individuals [83]. Young’s elastic modulus (YEM), a quantitative measurement of material stiffness, was increased in the common carotid with advancing age, quantitatively determining that arterial stiffening accelerates with advanced age. Clearly, aging has deleterious effects on the vasculature similar to hypertension and atherosclerosis, but it is an unavoidable risk factor.

### 4.1. Aging and Angiotensin II

Expression of the aforementioned genes and ensuing structural and cellular changes are mediated and accelerated by the pro-inflammatory effects of Ang II. Ang II induces signal transduction events which mimic the aged arterial phenotype with respect to arterial remodeling events. In one example, the aortas of young rats infused with Ang II displayed a gene expression profile akin to older arteries with increased expression of TGFβ and TGFβ-responsive genes such as matrix and matrix degrading enzymes [84]. These aortas also displayed histological changes such as intimal and medial thickening similar to much older rats. Small arteries were isolated from human subjects and examined histologically and functionally by myography [85]. Structurally, arteries from hypertensive individuals, regardless of age, resembled those of older individuals in terms of vascular remodeling, collagen deposition, and other fibrotic indices, all of which were compounded in aged hypertensive patients. Endothelium-dependent vasodilation was impaired in the hypertensive group and also correlated with the aged group. The authors also observed reduced nitric oxide availability in arteries from aged and hypertensive individuals, and concluded that oxidative stress in aged and hypertensive individuals was perhaps the driving force behind the increased vascular remodeling and reduced vascular reactivity. Furthermore, also in human subjects, the blockade of Ang II signaling not only reduced pro-inflammatory gene expression, but also delayed the development of age-associated aortic remodeling [68]. In one randomized clinical trial, elderly human subjects treated with the angiotensin receptor blocker Valsartan demonstrated improved age-related vascular compliance [66]. In one longitudinal study, carotid artery stiffness was correlated with advancing age [62], but addition of anti-hypertensive therapy was significantly correlated with improved artery distensibility, linking age, artery stiffness, and hypertension.

At the cellular and molecular level, many of the age-associated changes in the vasculature associated with Ang II can be attributed to the many deleterious effects of an increase in ROS. It is well accepted that oxidative stress can modify or otherwise irrevocably damage AND and other cellular machinery, indicative of many degenerative diseases, including hypertension, atherosclerosis, and especially ageing [86]. It has been shown in mice that disruption of AT1R prolongs longevity, which the investigators attributed to reduced ROS in the vascular wall [87]. These mice also developed reduced atherosclerosis as they aged. In other studies, Ang II-induced ROS induced an aging phenotype in VSMC, as assayed by increased replicative senescence, increased DNA damage, and a reduction in p53-mediated telomere length [82]. Together, these studies strongly suggest that the increased ROS induced by Ang II leads to enhanced VSMC biological aging. Consistent with the beneficial effects of AT2R engagement in terms of hypotension and attenuation of inflammatory signals, it was observed that over expression of the AT2R interacting protein ATIP, which regulates AT2R activity, reduced Ang II-induced VSMC senescence. Transgenic mice, which over express ATIP in aortic VSMC, demonstrated reduced senescence in thoracic aorta compared with control mice [88].

Similar to that observed in VSMC, one study demonstrated that Ang II induced senescent changes in EC indicative of the aging phenotype, including decreased proliferation, increased apoptosis, and abnormal cell morphology [89]. Considering that EC dysfunction is one of the earliest cellular harbingers of atherogenesis, this cellular event links Angiotensin II, aging, and atherosclerosis. Together, aging appears to predispose vascular cells, particularly VSMC, to an inflammatory phenotype and plaque development, and subsequently sets the stage for the more aggressive pro-inflammatory events initiated by atherogenic events such as sub-endothelial deposition of oxidized LDL.

### 4.2. Aging and Atherosclerosis

An unavoidable and unmodifiable risk factor for atherogenesis is advancing age. Expression of inflammatory cytokines, cell cycle proteins, and mitochondrial dysfunction have all been implicated as causative atherogenic events, and all are dysregulated in aged arteries. Experiments have been undertaken to determine if atherosclerosis can develop independently of chronic hyperlipidemia. At the systemic level, oxidative stress is increased with advancing age, as tissues from aged animals demonstrate increased ROS, which not only leads to increased oxidation of LDL, but age-related vascular remodeling similar to those described for Ang II-treated animals [90]. In one interesting study, it was found that increasing IL-6 levels correlated with mitochondrial dysfunction and mitophagy in aged versus young mice [91]. This study also showed that atherogenesis increased in aged wild-type mice, and hyperlipidemia further increased not only atherogenesis, but IL-6 expression and mitophagy. This study was confirmed by another group who observed that IL-6 and adhesion molecules were significantly increased in aorta from aged versus young mice [92]. Together, these studies indicate that aged VSMC are predisposed to a pro-inflammatory phenotype, and thus vulnerable to atherogenic stimuli. This also implicates inflammation and the pro-inflammatory cytokine IL-6 as a mediator of these effects. Another study in human atherosclerotic plaque determined that CAP VSMC expressed senescent marker gene expression that was absent in control arteries [93]. These markers correlated with shortened telomeres, the length of which was inversely proportional to the severity of atherosclerosis. The authors suggested that increased oxidative stress in proximity to the plaque drove the local VSMC into premature senescence.

## 5. Conclusions

The synthesis of the studies outlined in this review present an intimate relationship between hypertension, atherosclerosis, and aging. Chronic hypercholesterolemia induces the expression of many components of the RAS, and at multiple systemic and cellular targets, Ang II induces the expression of inflammatory and atherogenic proteins, which together leave the artery primed for hypertension and atherosclerosis. Inhibitors of Ang II synthesis and AT1R antagonists reduce vascular inflammation and plaque progression, and lipid-lowering therapy reduces blood pressure. In many murine studies, the genetic deletion of multiple components of the atherosclerotic pathway reduces hypertension, and deletion of members of the RAS attenuates atherogenesis. These studies are supported by clinical observations in humans using pharmacological inhibitors. Both of these diseases are more prevent in aged arteries, and their severity is exacerbated by age. Morphologically, advanced hypertensive and atherosclerotic arteries resemble those from aged individuals. Atherogenic and hypertensive stimuli intersect on the VSMC suggesting that hypertension, atherosclerosis, and vascular smooth muscle cells make a perfect trio for vascular pathophysiology. The identification of the molecular mediators of these diseases is key to our better understanding of them and, when supported by rigorous genetic studies in mice, represents an opportunity for the development of therapeutics to combat these vascular diseases which are approaching epic proportions.
